# Association Between Biomass Fuel Use and Depression Symptoms in the Adult Population of Oaxaca, Mexico

**DOI:** 10.3390/diseases13020047

**Published:** 2025-02-05

**Authors:** Roberto Ariel Abeldaño Zuñiga, Silvia Mercedes Coca, Moréniké Oluwátóyin Foláyan, Javiera Fanta Garrido, Gabriela Narcizo de Lima

**Affiliations:** 1 Institute of Research on Public Health, University of Sierra Sur, Oaxaca 70800, Mexico; scoca@unsis.edu.mx; 2 Helsinki Institute of Urban and Regional Studies (Urbaria), University of Helsinki, 00150 Helsinki, Finland; 3 Department of Child Dental Health, Obafemi Awolowo University, Ile-Ife 220101, Nigeria; toyinukpong@yahoo.co.uk; 4 Department of Clinical Sciences, Nigerian Institute of Medical Research, Lagos 100001, Nigeria; 5 National Institute of Social Services for Retirees and Pensioners, Buenos Aires 1043, Argentina; jfantagarrido@pami.org.ar; 6 Geography Department, Faculty of Arts and Humanities, Porto University, 4150-564 Porto, Portugal; gabrielalima@letras.up.pt; 7 Centre of Studies in Geography and Spatial Planning, Porto University, 4150-564 Porto, Portugal

**Keywords:** biomass, depression, gender disparities, rural areas, Oaxaca

## Abstract

Background: The impact of biomass fuel exposure on mental health, along with the associated gender disparities, remains largely unexplored. This study aimed to examine the association between biomass fuel use and depressive symptoms in the population of Oaxaca, Mexico, while also identifying gender differences in this relationship. Methods: This study used data from the 2022 National Health and Nutrition Survey (ENSANUT). Depressive symptoms, the outcome variable, were assessed using the Center for Epidemiologic Studies Depression Scale (CESD). The primary predictor variable was biomass fuel use, with gender, age, and residency stratum included as covariates. First, a binary logistic regression model was developed to estimate the dichotomous variable “depression symptoms”. Subsequently, a second binary logistic regression model was constructed to evaluate potential interactions between the covariates and the predictor variable. Findings: The sample included 1.4 million adults from Oaxaca, with a prevalence of depressive symptoms of 15%. Biomass fuel was used by 15.4% of the population. The first logistic regression model showed that women (Odds Ratio (OR): 1.249; 95% CI: 1.235–1.263; *p* < 0.001), individuals aged 60 years and older compared to the younger population group (OR: 12.192; 95% CI: 12.064–12.321; *p* < 0.001), those residing in rural areas (OR: 1.245; 95% CI: 1.232–1.259; *p* < 0.001), and individuals using firewood or charcoal for cooking (OR: 1.674; 95% CI: 1.651–1.697; *p* < 0.001) had higher odds of depressive symptoms. In the second binary logistic regression model, all associations and OR coefficients retained their direction, although the coefficients underwent a slight adjustment following the introduction of the interaction term, indicating the presence of an interaction. Conclusions: The study findings suggest a gendered association between biomass fuel exposure and depressive symptoms in the adult population of Oaxaca, with older women and women dwelling in rural areas being the most vulnerable. Interventions aimed at reducing biomass air pollution exposure and strengthening mental health support for women are strongly recommended.

## 1. Introduction

Gas emissions in urban environments are one of the primary triggers for respiratory diseases in urban areas [1,2]. However, pollutants in rural areas primarily originate from solid fuels, such as wood (firewood) and charcoal [3,4,5], used for cooking due to cultural practices or proximity to natural arboreal resources [6]. Although the use of solid fuel is recognized as a major cause of household air pollution leading to significant public health problems [7], the issue remains unresolved, particularly in communities where the most vulnerable populations reside [6].

The focus of studies on air pollution has been on the impact of ambient urban air on health in high-resource countries [8]. Little is known about the pattern of the impact of household air pollution on the health of rural communities in low- and middle-income countries [9]. Families are exposed to respirable particles of carbon monoxide, nitrogen and sulfur oxides, benzene, formaldehyde, 1,3-butadiene, and polycyclic aromatic compounds such as benzo[a]pyrene through household air pollution [7] resulting from the improper use of firewood as cooking and/or heating fuel [10]. This is of particular concern for women and children, who spend more time indoors. Women are often primarily responsible for cooking, increasing their exposure to biomass fuel-related pollutants [7,8,9,10]. This effect may accumulate over time, and the impact may worsen with age [11,12]. In rural areas of Mexico, biomass fuels such as firewood are often sourced from nearby forests, either through natural felling or the collection of deadwood, while charcoal is typically produced locally through small-scale traditional methods [13].

The production of formaldehyde during biomass fuel combustion, including wood and charcoal, is influenced by the chemical composition of the material and the combustion conditions. Wood, as a natural material, contains cellulose, hemicellulose, and lignin, which decompose during burning to release volatile organic compounds, including formaldehyde. Formaldehyde is formed during the pyrolysis phase of combustion when heat breaks down these organic components into smaller molecules under low-oxygen conditions [14,15].

In traditional cooking practices in rural settings, untreated wood is typically used as fuel, with no chemicals added after harvesting. However, the combustion process itself, particularly in open fires or traditional stoves with incomplete combustion, results in higher emissions of formaldehyde and other pollutants. This is compounded by local practices, such as using wood with varying moisture content or adding kindling materials that may affect combustion efficiency [16]. Studies have shown that these practices can increase the levels of formaldehyde and particulate matter in indoor air, contributing to household air pollution and associated health risks [14,15,16].

In rural Mexico, over half of rural households rely on these traditional fuels due to their affordability and cultural preferences, despite significant health and environmental implications [6]. The use of biomass in poorly ventilated spaces contributes to indoor air pollution, exposing families to high levels of particulate matter and increasing the prevalence of respiratory and cardiovascular diseases, particularly among women and children [17]. Environmental degradation is also a concern, as the unsustainable harvesting of wood for fuel leads to deforestation and soil erosion, exacerbating local climate vulnerabilities. Efforts to mitigate these issues have included introducing improved cookstoves, which are designed to reduce fuel consumption and emissions [6]. However, the adoption of these technologies has been uneven, influenced by socioeconomic barriers, cultural habits, and limited awareness of their benefits. Expanding access to cleaner energy alternatives, such as liquefied petroleum gas or renewable energy solutions, is essential for reducing reliance on biomass while improving health and environmental outcomes [17,18,19].

In Mexico, exposure to air pollution is linked to deaths due to cardiovascular, digestive, genitourinary, and respiratory diseases, with no evidence of effect modification by individual-level characteristics [20]. Studies focusing on household air pollution are scarce, descriptive, and tend to focus on specific or little communities. Oaxaca, Chiapas, and Guerrero have been identified as states with the highest usage of biomass fuel [6]. In addition, although the impact of household air pollution on the health of adults and neonates has been extensively studied elsewhere [10,21,22,23], little is known about its impact on mental health, and the differential impact on mental health by gender [24,25]. Some studies focus on the effects on cognitive decline and general mental well-being in the elderly population [23].

There is increased interest in the health effects of biomass fuel, but research on its link to mental health, particularly depression, is scarce. This scarcity creates significant gaps regarding the scope and mechanisms of the relationship. Long-term exposure to pollutants from biomass combustion, such as carbon monoxide, is associated with neuroinflammation and oxidative stress, which are implicated in the pathophysiology of depression. Other studies indicate that air pollutants (particulate matter, nitrogen oxides, and carbon monoxide) may increase the risk of psychosis, anxiety, and cognitive decline [24,25].

Currently, there is a lack of investigations into individual factors, such as gender, age, or pre-existing health conditions, that may influence the depression risk linked to biomass fuel use. Although the connection between biomass combustion and depressive symptoms is not entirely new, it remains relatively unexplored due to insufficient epidemiological evidence. Furthermore, it is estimated that the duration of exposure may be a critical factor, with prolonged exposure (lasting several years) potentially heightening the risk to mental health, including an increased risk of depression [26,27,28]. Moreover, chronic exposure may exacerbate health issues in vulnerable populations, particularly those with limited access to healthcare or in disadvantaged socio-economic conditions. Household air pollutants may, therefore, serve as a significant mental health risk factor for women, especially older women, who are more likely to spend extended periods indoors, continuously exposed to pollutants over time [25].

The study builds on the gender analysis framework, which examines how gender influences health outcomes and the social determinants of health [29]. This approach is particularly relevant when studying older women, as gender plays a critical role in exposure, vulnerability, and response to household air pollutants and mental health risks. A gender analysis would help in understanding how societal norms, gender roles, and caregiving responsibilities affect women’s exposure to household pollutants and their mental health, particularly in rural settings. The aim of this study, therefore, was to assess the association between the use of biomass fuels and depressive symptoms in the population of Oaxaca, Mexico, where the use of biomass fuel is significant [6]. The working hypothesis is that women with greater exposure to biomass fuels are at a higher risk of experiencing symptoms of depression.

## 2. Methods

### 2.1. Study Design and Study Population

This was a secondary analysis of data generated through the National Health and Nutrition Survey (ENSANUT) in Mexico in 2022 by the National Institute of Public Health of Mexico [30]. The purpose of ENSANUT is to obtain estimates of the health status, primarily focusing on risk factors for non-communicable chronic diseases in Mexican households [31]. The general objective of ENSANUT is to quantify the frequency, distribution, and trends of the population’s health and nutrition conditions and their determinants [31] to inform the design and evaluation of health policies and interventions in Mexico [30].

### 2.2. Sampling

ENSANUT utilized a multistage stratified sampling technique based on the National Sampling Frames constructed for population surveys [32]. ENSANUT has two types of sampling units for which estimates can be made: individuals (at the individual level) and households (at the aggregate level). The ENSANUT sample is nationally representative, and by applying longitudinal expansion factors (or weights) [30,31,32,33], estimates can be obtained for the entire population of the state of Oaxaca. After applying the sampling weights, the final sample in this study consisted of 1,406,132 adults aged 20 years or older, residing in the state of Oaxaca.

## 3. Dependent Variable

Prevalence of Depression Symptoms: The seven items from the Center for Epidemiologic Studies Depression Scale (CESD-7) [34] were used to estimate the prevalence of depressive symptoms. The CESD-7 scale evaluates the frequency of depressive symptoms in the week prior to the survey. Table 1 presents a complete breakdown of the questions, corresponding responses, and the scoring system used to screen for depressive symptoms [34]. After assigning the scores for each item, the total accumulated score for each adult is calculated, with a range of 0 to 21 points [34]. The cut-off points for moderate to severe depressive symptoms are 9 points for individuals aged 20 to 59 years and 5 points for individuals aged 60 years or older [34]. The lower cut-off for the elderly population reflects differences in symptom presentation with age; older adults often experience fewer affective symptoms but more somatic or cognitive manifestations, justifying a tailored threshold [35,36].

For conducting binary logistic regression analysis, depressive symptoms were categorized as positive symptoms (when scores were equal to or greater than the cutoff scores) and negative symptoms (when scores were less than the cutoff scores). The CESD-7 scale has been validated in the adult population of Mexico [34]. In the current study, the Cronbach’s alpha is 0.73.

### 3.1. Independent Variable

This exposure was determined from the variable ‘Fuel used for cooking in the household’. The response options to this question are firewood, charcoal, cylinder gas or stationary gas, natural gas or piped gas, electricity, other fuels, or do not cook. These seven options were dichotomized into: firewood and charcoal, considered as biomass fuels [6], and the remaining five options classified as ‘other fuels’. In this study, the exposure of interest (the primary independent variable) is biomass fuels (firewood and/or charcoal).

### 3.2. Covariates

The other independent variables in the study were gender (male, female) to account for differential exposure and vulnerability to biomass fuel-related pollution, age (categorized into two groups: individuals aged 20 to 59 years and individuals aged 60 years or older), and the level of urbanity of the residents’ locations. The rural stratum was considered for those residing in localities with fewer than 2500 inhabitants, while the urban or metropolitan stratum was assigned to those residing in localities or cities with 2500 inhabitants or more [30].

### 3.3. Data Analyses

The analysis utilized data from the 2022 National Health and Nutrition Survey (ENSANUT), which provided a weighted, population-representative sample of adults in Oaxaca, Mexico. The analysis considered the expanded sample (using the weights from the database) of adults aged 20 years or older residing in the state of Oaxaca, with complete data on the variables of interest. The raw data were first cleaned and merged from the ‘Adults’ and ‘Households’ datasets provided by ENSANUT. Sample weights were applied to ensure representativeness of the population, accounting for the multistage stratified sampling design.

Descriptive Statistics: Frequencies and percentages were calculated for all variables, stratified by depressive symptoms. Comparisons across groups were performed using chi-squared tests. The prevalence of depressive symptoms by age groups (20 to 59, and 60 and older) was calculated according to the methods followed by previous studies [34], Scores were dichotomized into positive and negative categories based on age-specific cutoffs (≥9 for ages 20–59 and ≥5 for ages 60+).

Subsequently, a preliminary binary logistic regression analysis model was defined to estimate the dichotomous variable ‘depression symptoms’ (y = 0, no depression; y = 1, depression). The covariates included the type of cooking fuel used, dichotomized as biomass fuels (firewood/charcoal) versus other fuels (e.g., gas, electricity), gender, age group (20–59 years, 60+ years), and residence stratum (rural vs. urban/metropolitan).

Finally, to verify whether there is an interaction between gender, residence stratum, and fuel used for cooking (gender × residence × fuel type), a second binary logistic regression model was defined, similar to the first, but with additional interaction terms between these three variables to address the heightened vulnerability of women in rural environments. These analyses are presented as odds ratios with their respective 95% confidence intervals. All analyses were conducted at a significance level of *p* < 0.05. SPSS v25 software was used to merge and analyze the ‘Adults’ and ‘Households’ databases from ENSANUT.

The selection of variables for the logistic regression models was guided by both theoretical and practical considerations [37,38], prioritizing parsimony to ensure a focused and interpretable analysis. We included age, rural residence, gender, and cooking method as predictors based on their established relevance to the research aim and their potential to capture the key determinants of depressive symptoms in the context of biomass fuel use. This parsimonious approach was designed to minimize the risk of overfitting and to focus on the gender perspective. While additional variables, such as social class and physical illness, are recognized as important determinants of depression in the broader literature, they were excluded to maintain the model’s simplicity. The focus of this study is on the specific relationship between the selected covariates and depressive symptoms, acknowledging that further research with more comprehensive datasets could explore additional factors.

### 3.4. Ethical Considerations

This study utilized publicly available secondary data obtained from the website of the National Institute of Public Health of Mexico [30].

## 4. Results

Table 1 presents the data for 1,406,132 individuals extracted from the EN-SANUT dataset. Of these, 850,142 (60.4%) were female, 792,417 (56.3%) resided in rural areas, and 293,144 (20.8%) were aged 60 years or older. Additionally, 211,223 (15.0%) showed depressive symptoms, and 217,077 (15.4%) used biomass fuel (firewood or charcoal). A significantly higher proportion of women reported depressive symptoms compared to men (16.7% vs. 12.5%; *p* < 0.001). Furthermore, a greater percentage of individuals who lived in rural areas compared to urban or metropolitan regions reported symptoms of depression (17.1% vs. 12.3%; *p* < 0.001), and a higher proportion of those using biomass fuels showed depressive symptoms compared to those using other types of fuel (22.0% vs. 13.7%; *p* < 0.001). Finally, individuals aged 60 years or older had a significantly higher prevalence of depressive symptoms than those aged 20 to 59 years (47% vs. 6.6%; *p* < 0.001).

Table 2 indicates that women had significantly higher odds of experiencing depressive symptoms compared to men (Odds Ratio (OR): 1.249; 95% Confidence Interval (CI): 1.235–1.263; *p* < 0.001). Additionally, individuals aged 60 years and older had significantly higher odds of depressive symptoms compared to the younger population group (OR: 12.192; 95% CI: 12.064–12.321; *p* < 0.001). Those residing in rural areas also had significantly higher odds of experiencing depressive symptoms compared to those living in urban and metropolitan areas (OR: 1.245; 95% CI: 1.232–1.259; *p* < 0.001). Furthermore, individuals who used firewood and charcoal for cooking had significantly higher odds of depressive symptoms compared to those who used other types of fuels (OR: 1.674; 95% CI: 1.651–1.697; *p* < 0.001).

Furthermore, Table 2 demonstrates that the coefficients underwent a slight adjustment following the introduction of the interaction term (gender * residency stratum * type of cooking fuel). Individuals using biomass fuel had significantly higher odds of experiencing depressive symptoms compared to those using other fuels (OR: 1.420; 95% CI: 1.384–1.458; *p* < 0.001), indicating the presence of an interaction between these variables.

## 5. Discussion

The results of the current study provide the first population-based epidemiological evidence on the association between exposure to biomass fuels and depressive symptoms in the adult population of Oaxaca. The findings suggest that nearly 1 in 7 individuals in Oaxaca experienced depressive symptoms in 2022. Among those with depressive symptoms, over 60% were female, resided in rural areas, were aged 60 or older, and used solid fuels. Individuals using biomass fuel had significantly higher odds of experiencing depressive symptoms.

One of the strengths of this study lies in its use of a large, population-based dataset (ENSANUT 2022), which provides a representative sample of over 1.4 million adults in Oaxaca. The large sample size enhances the credibility and applicability of the findings within the state’s context. Additionally, the study utilizes a validated screening tool for depressive symptoms (CESD-7), ensuring consistent measurement of mental health outcomes. By incorporating gender, age, and residence stratum as covariates and examining interaction effects, the analysis offers insights into the varying impact of biomass fuel use on mental health, particularly among vulnerable groups such as rural women. Furthermore, the study explores the intersection of environmental and mental health risks, a relatively underexplored area in public health, particularly in low- and middle-income countries. Its focus on the gendered implications of household air pollution makes a significant contribution to the field, providing valuable guidance for future interventions and policy development.

The 15% prevalence of depression observed in the Oaxacan population is consistent with the 16.7% prevalence found in another study using the same data source across Mexico [34]. The current study in Oaxaca confirms that women are at a higher risk of depressive symptoms compared to men, aligning with previous research [23,24,25]. However, the most novel aspect of this study is the demonstrated interaction between this risk, the use of biomass fuels, and residence in rural areas. The gendered nature of this risk is evident not only through women’s higher exposure to indoor air pollution but also through their traditional caregiving roles, which often keep them confined to domestic spaces where they are disproportionately exposed to harmful fuels. Additionally, the risk appears to increase with age, likely due to the duration of exposure, as other studies using longitudinal designs have suggested [23]. This heightened risk may be tied to women’s higher exposure to biomass air pollution resulting from their domestic and caregiving duties, a finding consistent with prior studies [21,23,24,25]. Therefore, interventions aimed at reducing exposure to biomass air pollution and improving mental health should address the specific needs and experiences of women [25].

The mechanisms by which exposure to compounds derived from biomass combustion is linked to mental disorders have been explored in previous studies [39]. Research in rodents has shown associations between exposure to volatile organic compounds and depression [39]. Notably, increased concentrations of benzene were linked to a rise in central nervous system symptoms [39]. Additionally, exposure to benzene and m-xylene led to impaired learning ability, motor disorders, and anxiety-like behaviors in rodents [39].

The findings have significant implications for public health and policy, emphasizing the need for measures to reduce exposure to biomass air pollution and improve mental health, particularly among vulnerable groups such as women in rural areas. From a gender perspective, nature-based solutions, such as clean biofuels, play a crucial role in alleviating the health risks associated with biomass exposure that disproportionately affect women. These solutions offer a promising mitigation strategy to reduce women’s exposure to harmful air pollution within their homes [40]. Additionally, beyond the health problems caused by exposure to indoor air pollution, these issues contribute to the perpetuation of poverty cycles. Although the economic aspect was not directly included among the study variables, it is important to consider the context of Oaxacan women. Women from low-income backgrounds often face a dual burden of health and economic challenges. They experience greater exposure to pollutants and incur higher out-of-pocket healthcare expenses due to the increased incidence of non-communicable chronic diseases associated with biomass fuel use. This combination of heightened health risks and financial strain further entrenches poverty and diminishes their overall quality of life [8,10,23].

Similarly, there is evidence that frames household air pollution not only as a health issue but also as a form of gender injustice, environmental injustice, and economic inequality [40]. The quality of the air within homes is intricately linked to socioeconomic disparities. Poor indoor air quality, resulting from the fuels used for cooking, predominantly affects low-income households in rural areas and women, who experience higher exposure due to their greater involvement in household activities. Indoor air pollution tends to concentrate in regions where cleaner fuels, such as natural gas, are inaccessible, often in areas populated by the most disadvantaged communities. Women, particularly those in rural and low-income environments, shoulder the brunt of this environmental injustice, which exacerbates existing gender and economic inequalities. Therefore, ambient air pollution in homes is not only an environmental issue but also a social and economic one, as the impacts of environmental degradation are neither uniform nor equitably distributed [21,23,25].

Several limitations of this study should be acknowledged. First, the study did not directly measure household pollution levels but instead relied on the type of fuel used for cooking as a proxy for exposure. While the type of fuel can provide an indication of potential exposure, it does not capture the actual concentration of harmful chemical compounds in the air. Second, the cross-sectional design of the ENSANUT survey limits the ability to establish causal relationships; the analysis can only identify associations between the variables. This design does not account for the temporal dynamics of exposure or the potential for changes in pollutant levels over time. Third, the study was unable to explore the interaction of biomass fuel use with other comorbidities, such as respiratory or cardiovascular diseases, which are known to be associated with biomass fuel exposure, as discussed in the introduction [21,23]. Moreover, future studies that collect primary data could provide insights into the practices, sources of biomass fuels, and their potential implications for exposure levels and associated health risks. Lastly, the CESD-7 scale does not provide a clinical diagnosis of depression; rather, its results are only a screening tool for symptoms indicative of depression [34]. However, it is a validated scale and is widely used for screening purposes.

It is important to acknowledge that using age-specific cut-off points for depressive symptoms may affect the observed relationships, particularly when analyzing age effects. Future research should explore alternative measures of depression, such as clinical indicators, specific symptom domains (e.g., behavioral or somatic), or empirically derived thresholds that remain consistent across age groups, to validate and build upon the findings of this study. Additionally, the higher prevalence of depressive symptoms observed among older adults in our study is consistent with the existing literature [41,42,43], which indicates that aging-related processes significantly increase vulnerability to depression. Biological factors, such as changes in brain structure [43] (including reductions in hippocampal volume and frontostriatal integrity) may contribute to the increased risk of depression in older individuals. Cognitive decline and chronic health conditions, which are more prevalent in older populations, have also been identified as significant contributors to depression, as they increase physical and emotional burdens, exacerbate feelings of isolation, and reduce functional independence [42]. Furthermore, psychosocial adversities, such as economic insecurity, bereavement, caregiving responsibilities, and social isolation, further exacerbate the risk of depression in older adults [41].

These factors can interact with biological vulnerabilities, creating a multifaceted risk profile that necessitates tailored intervention strategies. Despite this complexity, it is worth noting that healthy, well-functioning older adults without chronic illnesses do not appear to have a significantly higher risk of depression compared to younger adults. Therefore, addressing the interplay between chronic health conditions, cognitive impairment, and psychosocial stressors is critical for understanding the age-related differences in depression and for developing targeted prevention and treatment strategies. Recognizing these distinctions is essential for refining diagnostic tools and interventions. Future studies should explore the age-dependent mechanisms of depression, including the interaction of biological, cognitive, and psychosocial factors, using diverse measures of depressive symptoms to capture the full spectrum of this heterogeneous condition across the lifespan.

Although further research is needed to understand the mechanisms underlying the associations identified in this study, its strengths lie in the suggestion that exposure to biomass fuels could be an important risk factor for depression in the adult population of Oaxaca. It is crucial to incorporate a gendered perspective into public policies and interventions aimed at reducing exposure to biomass-related air pollution, given its disproportionate impact on women. Public policies and interventions should be implemented to mitigate exposure to biomass-related air pollution and improve mental health, especially among vulnerable groups such as women living in rural areas.

Based on the results of this study, the following recommendations are made. First, longitudinal studies are recommended to confirm the causal relationship between exposure to biomass-related air pollution and depression [28,44]. Additionally, it is recommended to develop and implement interventions aimed at reducing household exposure to biomass-related air pollution, particularly in rural areas. Mental health promotion and depression care programs should be strengthened, with special attention to women living in rural areas, as mental health services may be distant from their residences [45]. Finally, it is strongly recommended that a gender-sensitive approach be integrated into all public policies and interventions addressing both environmental and mental health [46]. By doing so, policies can more effectively address the systemic inequalities that place women—especially those in rural and low-income areas—at a higher risk of environmental and mental health issues [47,48]. This approach ensures that efforts to improve health outcomes are inclusive and tackle the underlying gender disparities that exacerbate these risks, promoting a more equitable distribution of resources and support. Moreover, recognizing the principles of environmental justice within this framework is crucial, as it seeks to rectify the disproportionate impacts of environmental effects on marginalized communities, fostering equitable access to healthy environments for all women [40].

## 6. Conclusions

The overall prevalence of depressive symptoms in the adult population of Oaxaca in 2022 was 15%, with women (16.7%) showing a higher prevalence than men (10.3%). A significant association was found between the use of biomass fuels and the prevalence of depressive symptoms. Individuals using firewood or charcoal for cooking were 1.67 times more likely to experience depressive symptoms compared to those using other types of fuels. This association remained significant even after adjusting for factors such as gender, age, area of residence, and other socioeconomic indicators. Furthermore, a significant interaction was identified between gender, area of residence, and the type of fuel used for cooking. Women living in rural areas and using firewood or charcoal for cooking were at the highest risk of developing depressive symptoms.

This study contributes to the understanding of how environmental exposures, such as household air pollution from biomass fuel use, intersect with gender and rurality to influence mental health outcomes. This intersectional approach fills a gap in the literature by focusing on the mental health impacts of biomass fuel use, which has been understudied compared to physical health outcomes. These findings are crucial for designing targeted interventions that address the specific needs and experiences of women in this context. Moreover, the identified interaction underscores the importance of tackling gender and socioeconomic disparities to improve the mental health of this population group. Adopting a gender-sensitive approach is essential for the effective design and implementation of interventions. Women, especially those in rural and low-income areas, require targeted support, including access to affordable clean energy alternatives, mental health services, and social programs that alleviate caregiving burdens. By addressing the intersecting challenges of environmental and mental health inequities, these efforts could not only improve individual health outcomes but also promote broader social and economic equity within vulnerable populations.

## Figures and Tables

**Table 1:** Prevalence of depressive symptoms by gender, residence stratum, and fuel used for cooking. Oaxaca, 2022.

Variables	Categories	Depression Symptoms				Total (100%)	*p* Value
		Positive		Negative			
		*n* (211,223)	% (15.0)	*n* (1,194,909)	% (85.0)		
Gender	Females	141,948	16.7	708,194	83.3	850,142	<0.001
	Males	69,275	12.5	486,715	87.5	555,990	
Residence Stratum	Urban or metropolitan	75,474	12.3	538,241	87.7	613,715	<0.001
	Rural	135,749	17.1	656,668	82.9	792,417	
Cooking Fuel	Firewood or Charcoal	47,804	22.0	169,263	78.0	217,067	<0.001
	Other fuels	163,419	13.7	1,025,646	86.3	1,189,065	
Age Groups	20 to 59	73,454	6.6	1,039,534	93.4	1,112,988	<0.001
	60 and older	137,769	47.0	155,375	53.0	293,144	

Source: Elaborated by the authors using data from ENSANUT 2022.

**Table 2:** Odds Ratio for risk indicators associated with depressive symptoms in Oaxaca in 2022.

	Model 1 Without Interaction				Model 2 with Interaction			
Variables (Categories)	Odds Ratio	Confidence Interval 95%		*p* Value	Odds Ratio	Confidence Interval 95%		*p* Value
		Lower Limit	Upper Limit			Lower Limit	Upper Limit	
Gender (females)	1.249	1.235	1.263	<0.001	1.170	1.156	1.184	<0.001
Age group (60 and more)	12.192	12.064	12.321	<0.001	12.190	12.063	12.319	<0.001
Residence Stratum (rural)	1.245	1.232	1.259	<0.001	1.226	1.212	1.240	<0.001
Cooking fuel (firewood or charcoal)	1.674	1.651	1.697	<0.001	1.423	1.397	1.450	<0.001
Interaction: Gender × residence stratum × cooking fuel	-	-	-	-	1.420	1.384	1.458	<0.001

Source: Elaborated by the authors using data from ENSANUT 2022.

## Data Availability

This study utilized publicly available secondary data obtained from the website of the National Institute of Public Health of Mexico at: https://ensanut.insp.mx/ (accessed on 2 September 2024).
